# A case of endobronchial actinomycosis as a challenging cause of lung atelectasis

**DOI:** 10.1186/s12879-021-06126-3

**Published:** 2021-05-08

**Authors:** Nicoletta Golfi, Roberta Mastriforti, Luca Guidelli, Raffaele Scala

**Affiliations:** 1 Pulmonology and RICU, S Donato Hospital, Arezzo, Italy; 2 Internal Medicine, S Sepolcro Hospital, Arezzo, Italy

**Keywords:** Bronchoscopy, Endobronchial actinomycosis, Endoscopic treatment, Lung atelectasis

## Abstract

**Background:**

Primary endobronchial actinomycosis is exceptionally uncommon and can be misdiagnosed as unresolving pneumonia, endobronchial lipoma, bronchogenic carcinoma or foreign body. Predisposing factors are immunosuppressive conditions, chronic lung diseases, poor oral hygiene or foreign body aspiration.

**Case presentation:**

We reported a case of 88-year old woman with a 4 days history of mild exertional dyspnea, productive cough with purulent sputum and fever up to 37.8 °C, who developed left sided endobronchial actinomycosis in absence of any pre-existent risk conditions; endobronchial de-obstruction and specific antibiotic treatment were performed with success, achieving a full resolution of the disease, with bronchoscopy playing a key role in the diagnosticand therapeutic pathways.

**Conclusions:**

This case raises the necessity for increased awareness in the management of endobronchial lesions and in cases of suspected endobronchial actinomycosis; bronchoscopy plays a key role in the diagnostic and therapeutic process; prompt recognition of this entity can expedite proper treatment and recovery.

## Background

Actinomycosis is a rare, chronic, suppurative infection due to *Actinomyces spp.*, a group of Gram positive, anaerobic or microaerophilic bacteria, belonging to human resident flora of the oropharynx and gastrointestinal tract. It is estimated that pulmonary form of actinomycosis constitutes approximately 15 to 45% of the total burden of the disease and common primary lung lesions involve peribronchial tissue, bronchioles and alveoli, often from aspiration of orapharyngeal or gastrointestinal secretions [1]. Risk factors are usually immunosuppressive conditions, chronic lung diseases, poor oral hygiene or foreign body aspiration. Pulmonary actinomycosis is often misdiagnosed as clinical presentation is heterogeneous, including cough, fever, hemoptysis and weight loss. In addition, imaging findings present low specificity, making the diagnosis challenging, as differential diagnosis include other diseases, such as lung cancer, tuberculosis, pneumonia and aspergillosis [2]. In this case, pulmonary actinomycosis was presented with endobronchial involvement in the absence of any predisposing conditions, mimicking other diseases such as unresolving pneumonia, endobronchial lipoma, bronchogenic carcinoma or foreign body. Diagnosis was confirmed with bronchoscopic biopsies; endoscopic treatment and long-term antibiotic therapy turned out to be successful.

## Case presentation

**Fig. 1:**
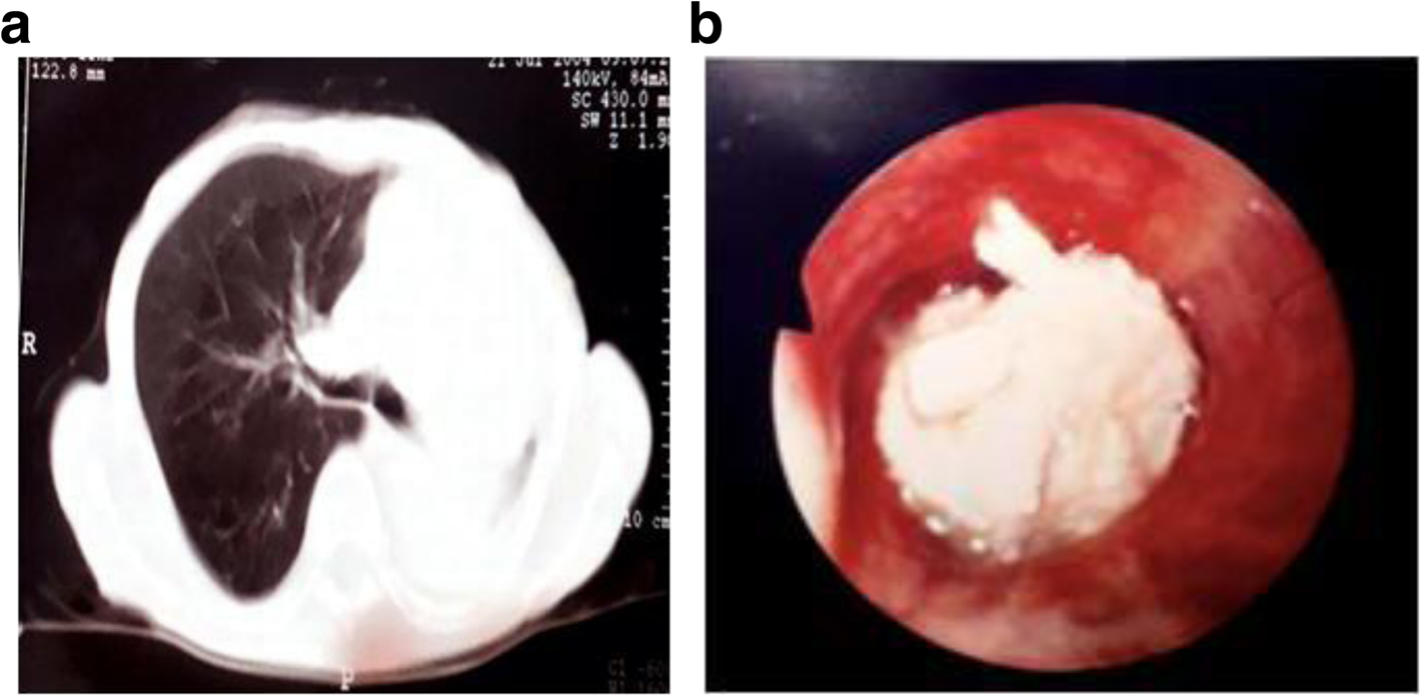
**a** Chest CT scan revealing a complete left atelectasis by endobronchial mass. **b** Bronchoscopic finding demonstrating the presence of an endobronchial white necrotized mass causing full occlusion of left main bronchus

A 88-year old woman was admitted to the emergency department of peripheral hospital with a 4 days history of mild exertional dyspnea, productive cough with purulent sputum, fever up to 37.8 °C and asthenia. Her past medical history consists of Chron’s disease (treated with hemicolectomy and pharmacological therapy with mesalamine) and osteoporosis, with left femoral arthroplasty occurred in the previous month. At admission the patient’s oxygen saturation was 96% and body temperature was 37.8 C. Patient’s blood pressure was 95/60 mmHg. Chest auscultation showed a marked reduction of lung sound in left hemithorax associated with bronchial breath. Chest X-ray revealed a complete left atelectasis, which was confirmed by Computed Tomography (Fig. 1a). Laboratory findings showed a normal leukocyte count (5.9 × 10^6^/L), a high level of erythrocyte sedimentation rate (ESR) [101 mm/h] and an elevated fibrinogen serum level (627 mg/dl). Due to clinical presentation, laboratory and imaging findings, the presence of mucus plug or foreign body was suspected at first; antibiotic treatment with ampicillin 1 g and sulbactam 2 g twice a day and methylprednisolone 20 mg twice a day was empirically started. The patient underwent flexible bronchoscopy under mild analgo-sedation (midazolam 3 mg i.v, fentanyl 50 mcg i.v) which revealed an endobronchial white necrotized lesion fully obstructing left main bronchus (Fig. 1b): multiple bronchoscopic biopsy specimens for histopathologic and microbiologic analysis were taken; mechanical and laser-assisted removal of the mass was performed by means of jumbo forceps with partial airway re-canalization. Histopathologic findings obtained by the examination of biopsy specimens showed fragments of the bronchial wall with signs of chronic exudative inflammation and dysplasia of the lining epithelium; furthermore, there were radiating filamentous colonies consistent with actinomyces growth. No extra-bronchial and pulmonary localization of actinmoycotic disease was detected by total body CT scan. The patient was discharged after 1 week and antibiotic treatment was switched to levofloxacin 500 mg once a day for another week with the indication to perform another bronchoscopy after 2 weeks. New bronchoscopic findings demonstrated the huge reduction in the size of the mass, which was present only in the third lower part of the left main bronchus without signs of obstruction. This residual mass was removed once again with laser-assisted technique. A third bronchoscopy was performed 20 days later and demonstrated the complete re-absorption of the lesion. Concomitantly, there was almost full improvement in symptoms.

## Discussion and conclusions

Actinomycosis is an infectious disease due to *Actinomyces spp.,* a group of Gram positive, anaerobic or microaerophilic bacteria, belonging to the resident flora of the oropharynx and gastrointestinal tract. The disease is classically divided into three types according to the anatomic sites involved: cervicofacial, abdominopelvic and thoracic; in particular, thoracic forms represent 15 to 45% of total cases. Aspiration of organism from the oropharynx is the usual source of thoracic actinomyces infection. Chronic lung diseases, such as bronchiectasis, alcoholism, diabetes, hematologic diseases, human immunodeficiency virus infection and the use of immunosuppressive agents have been associated with the development of pulmonary actinomycosis. Furthermore, in cases of endobronchial development of actinomycosis, broncholithiasis and endobronchial foreign bodies seem to represent predisposing factors [3].

The peculiarity of the reported case is the lack of any known pre-existent risk factors such as history of immunosuppression and of chronic lung diseases, poor oral hygiene or foreign body aspiration.

The suspect of pulmonary actinomycosis is usually raised by radiologic abnormalities: atelectasis due to endobronchial mass, interstitial and/or pleural thickening could be depicted by chest CT scan [4]. However, diagnosis of pulmonary actinomycosis is often challenging because of the absence of specific symptoms and radiological features: a wide range of CT findings have been described in pulmonary actinomycosis, such as air-space or lobar consolidation, ground glass opacities, pleural effusions, pleural thickening, hilar lymphadenopathy and necrotic mass [5]. Bronchoscopic findings of endobronchial actinomycosis are also no specific, as it could appear as a wall thickening with partial occlusion of bronchi or a submucosal or exophytic mass, sometimes with necrotic parts, leading to partial or full bronchial obstruction, simulating lung cancer or tuberculosis [6–8]. That is the reason why actinomycosis endobronchial involvement has to be taken into account in the differential diagnosis of an endobronchial mass [9], which includes benign (hamartoma, lipoma and fibroepithelial polyp) and malignant endobronchial lesions (bronchial neuroendocrine tumors, bronchogenic tumors, endobronchial metastasis or mucoepidermoid carcinoma) as well as infective endobronchial lesions (endobronchial tuberculosis or *Nocardia*) and miscellaneous causes (mucus plug or foreign body).

Bronchoscopy plays a key role in the diagnostic process: indeed, the histopathological examination of tissue specimens obtained during bronchoscopic procedures is essential to make a diagnosis of endobronchial actinomycosis. Histopathologic findings are characterized by the presence of chronic and exudative inflammation signs, presence of polymorphonucleates and fibroblasts; in particular sulphur granules and radiating filamentous colonies showed at hematoxylin and eosin staining is highly suggestive of actinomycosis [10]. Bronchoscopy is effective in removing the endobronchial tissue and therefore re-canalize the obstructed airways. Even if rigid bronchoscopy represents the best option for the safe management of airway stenosis, in our case we were successful to re-open the obstructed left main bronchus by means of jumbo forceps during flexible bronchoscopy under laser assistance. Softness of the tissue and good respiratory conditions may have favoured this approach helped by mild analgo-sedation while the patient was on spontaneous breathing.

Prognosis is usually favourable and treatment is based on prolonged antibiotic therapy, such as intravenous penicillin G (18–24 million units daily) for 2-6 weeks, followed by oral penicillin V for 6–12 months as recommended treatment. In addition, treatment with β-lactams, such as amoxicillin and ceftriaxone, doxycycline and clindamycin have often been successfully used, as in this specific case was used ampicillin [10]. Despite the recommendations for prolonged treatment, many patients have been cured with less than 6-months of antibiotic therapy [11]. Bronchoscopic follow up is of relevant importance to exclude the presence of foreign body, that could only be detected after the start of an antibiotic treatment and has to be removed, by mechanical or laser assisted technique.

A key point is to early recognize and treat endobronchial actinomycosis in order to avoid dangerous consequences such as rib destruction, mediastinal complications which could lead to involvement of heart and fatal pericarditis [12].

Endobronchial actinomycosis is a rare infectious disease whose diagnosis may be challenging as it may simulate other more common conditions such as unresolving pneumonia, lung cancer, endobronchial lipoma, bronchogenic carcinoma, foreign body, endobronchial tuberculosis [9]. This issue raises the necessity for increased awareness in the management of endobronchial lesion and in cases of suspected endobronchial actinomycosis; bronchoscopy plays a key role in the diagnostic process as it can provide specimens for histopathologic examination and anaerobic cultures that are required in order to make the final diagnosis.

## Data Availability

All data generated or analysed during this study are included in this published article.

## Abbreviations

CT: Computed Tomography
ESR: Erythrocyte Sedimentation Rate
